# Does Spiritual Intelligence (SI) Exist? A Theoretical Investigation of a Tool Useful for Finding the Meaning of Life

**DOI:** 10.1007/s10943-020-01005-8

**Published:** 2020-02-27

**Authors:** Katarzyna Skrzypińska

**Affiliations:** Institute of Psychology, University of Gdańsk, Bażyńskiego 4, 80-309 Gdańsk, Poland

**Keywords:** Spirituality, Spiritual intelligence, Meaning of life, Satisfaction with life, Well-being

## Abstract

For years, spirituality and finding the meaning of life have been considered essential phenomena in the context of human existence. Zohar introduced the term *spiritual intelligence* (SI) in 1997, and since that time researchers have been seeking to clarify the concept. Emmons (The psychology of ultimate concerns. Guilford Press, New York, 1999) suggested that SI serves as a potentially significant construct to expand our understanding of the psychological determinants of human functioning. In recent years, several efforts to conceptualize and measure this construct have joined the body of related literature, of which King (Brighter paths to wellbeing: an integrative model of human intelligence and health. Trent University Centre for Health Studies Showcase, pp 12–13, 2008) serves as one notable example. Following, evaluating, and summarizing the theoretical debate regarding the validity of a concept, as it is presented in the literature, has long been understood as a helpful way of extending scholarly dialogue. In this project, I review psychological literature relevant to the debate on the validity of SI as a psychological construct. The literature offers many examples that demonstrate a relation between SI and other phenomena that are important for human functioning—well-being in this. Results of the analysis support theoretical considerations for viewing SI as facilitating the ability to search for the meaning of life and provide directions for future study.

Although spirituality has been the domain of theology and philosophy for ages, only in the last century has it become a recognized topic of study within the discipline of psychology. Spirituality is now widely investigated in the psychological literature, especially in relation to several other phenomena, such as personality (Emmons 1999; MacDonald 2000), searching for the meaning of life (Park 2005), coping (Pargament 1997), well-being (Emmons 1999), and health (Koenig 1997, 2011). Sisk (2019) suggests that spirituality may also have properties best understood in terms of special abilities. Yet, spirituality, as a theoretical construct, remains poorly understood. Fortunately, scholars around the world continue their efforts to study and define it (Hood et al. 2009; Oman 2013; Skrzypińska 2014, etc.).

Many spiritual traditions (e.g., Buddhism, shamanism, spiritualism, etc.) and also portions of the psychological literature (e.g., Baumeister 1991; Wulff 1997; Zohar and Marshall 2000) emphasize both transformation of consciousness and finding meaning as important factors in spiritual development (see also Frankl 1966; Park 2005; King 2010; Skrzypińska 2014; Sisk 2019). In order for these processes to work, spirituality requires an instrument to facilitate, or enable, many of its activities (especially the search for both general and personal meaning in life). Any transformation of consciousness also requires a medium to achieve its goals (Emmons 1999; Sisk 2019). In this project, I evaluate the much debated concept of *spiritual intelligence* (SI) as a specific phenomenon that might serve as the required instrument or medium for these processes. Additionally, I seek to explain how SI may relate to the larger concept of spirituality, and investigate potential relations between SI and other phenomena, such as personality, the search for the meaning of life, and well-being.

## Definitions and Models of Spiritual Intelligence

The most common term for intelligence (in general) is to understand it as the degree to which one can adapt to one’s environment (https://allpsych.com/dictionary/i/). Moreover, Gardner (2000) states that intelligence is largely inborn and therefore difficult to alter. It follows that psychologists can measure intelligence, from early in a subject’s life through the administration of circumscribed instruments called IQ tests.

As a result of the growing body of research on spirituality in general (Hood et al. 2009; Oman 2013; Paloutzian and Park 2013; Streib and Hood 2016) and works on the topic of spiritual intelligence (SI) in particular (e.g., Amram 2007; Halama and Striženec 2004; Mayer 2000; Noble 2000; Zohar and Marshall 2000), many conceptualizations of this construct have emerged (e.g., Emmons 1999; Gardner 1999; Amram 2007; King 2008; Griffiths 2017). Zohar introduced the term *spiritual intelligence* in 1997. Emmons (1999) describes it as an instrument of mature personality that enables the fulfillment of spiritual goals or strivings (see also Zohar and Marshall 2000; Halama and Striženec 2004). Following this line of thinking, SI—at the appropriate level of self-consciousness and wisdom—may facilitate a person’s search for meaning in life and may aid them in achieving complex spiritual goals (e.g., conversion, dealing with a crisis, obtaining salvation, etc.).

Some scholars, such as Emmons (1999, 2000a), claim that SI is a form of intelligence involving a set of capacities and abilities that enables people to solve problems and attain goals in their everyday lives. This definition assumes that spirituality may be conceptualized in adaptive, cognitive–motivational terms. Following this line of thinking, SI consists of a number of abilities and competencies that may be part of a person’s expert knowledge and that are relevant in problem-solving situations.

Emmons (2000a) identified five components of SI:

- the capacity to transcend the physical and material;
- the ability to experience heightened states of consciousness;
- the ability to sanctify everyday experience;
- the ability to utilize spiritual resources to solve problems; and
- the capacity to be virtuous.

In response to Mayer’s (2000) criticism of the last component, which refers to ethics and personality rather than to intelligence, Emmons dropped it from his revised model, retaining only the first four components (Emmons 2000b).

Emmons (1999) claimed that, according to Gardner’s theory of multiple intelligences, SI meets the criteria for an independent intelligence modality (see below for further discussion). Gardner (2000), however, did not agree, and suggested *existential intelligence* (ExI) as a feasible alternative to SI (Gardner 1993, 1999). ExI is understood by Halama and Striženec (2004) as the ability to develop a system of beliefs and values that allows a person to recognize the existential meaning of life and the existential meaning of every situation. Unfortunately, this definition is rather general and difficult to operationalize. Although Gardner suggests ExI as an alternative to SI, Gardner, himself, did not include it in his earlier (1993) model due to the lack of quantifiable scientific criteria (Gardner 2000). Gardner maintains that both theoretical and practical limitations make SI a highly controversial construct in psychological literature.

According to the sources cited above, SI is understood as an ability to solve problems, seek meaning, and express values (Zohar and Marshall 2000), but so too is ExI (Halama and Striženec 2004). At first glance, SI and ExI appear to be very similar, if not duplicate constructs, but looking deeper, ExI seems to be broader notion than SI (Skrzypińska 2008). SI functions within more narrow parameters to control more detailed and specialized action than ExI does, especially with regard to beliefs and values. Zohar (1997, 2004) visualizes SI as an aspect of intelligence that sits at the conscious level of meaning and purpose—above the traditional measure of IQ and the various notions of *emotional intelligence* (EI). For Zohar, SI is derived from the properties of a living, complex, adaptive system. Emmons (2000a) presents a similar point of view by claiming that SI qualifies as a distinct intelligence modality. His particular definition specifies it as a “the adaptive use of spiritual information to facilitate everyday problem solving and goal attainment” (p. 176). He further indicates that SI sensitizes a person to transcendental reality and offers her the possibility of searching for “unity” to realize her highest potential (Emmons 1999). In light of the above characteristics, a distinction between existential and spiritual intelligences seems reasonable. To summarize the relationship between SI and ExI as described in the psychological literature, I emphasize that (a) SI is not the same as ExI is, but concerns the same tendency, i.e., looking for meaning; (b) SI is narrower aspect of ExI; (c) ExI develops a need and perspective for the search for existential meaning, but SI delivers specific tools for actually doing the searching.

Griffiths (2017) defines SI as a higher dimension of intelligence that activates the qualities and capabilities of the authentic self in the form of wisdom, compassion, integrity, joy, love, creativity, and peace. According to Griffiths, SI results in a deeper sense of meaning and purpose. Additionally, SI enhances a wide range of important skills (both life skills and work skills). Griffiths understands SI to be a consequent effect of the presence and action of both the intellectual and the emotional intelligence modalities. In this reasoning, Griffiths’ approach differs from Emmons’ and from Halama and Striženec’s. Griffiths’ definition of SI is not as focused and precise as theirs are; he understands SI as a higher dimension of intelligence in general.

King (2008) takes a more empirical approach. He defines SI as a “set of mental capacities which contribute to the awareness, integration, and adaptive application of the nonmaterial and transcendent aspects of one’s existence, leading to such outcomes as deep existential reflection, enhancement of meaning, recognition of a transcendent self, and mastery of spiritual states” (p. 56). Working from this definition, King created his Spiritual Intelligence Self-Report Inventory (SISRI-24), for which he developed the four original scales identified below:

- Critical Existential Thinking (CET)—the capacity to critically contemplate meaning, purpose, and other existential/metaphysical issues (e.g., existence, reality, death, the universe); to come to original existential conclusions or philosophies; and to contemplate nonexistential issues in relation to one’s existence (i.e., from an existential perspective);
- Personal Meaning Production (PMP)—the ability to derive personal meaning and purpose from all physical and mental experiences, including the capacity to create and master (i.e., live according to) a life purpose;
- Transcendental Awareness (TA)—the capacity to identify transcendent dimensions/patterns of the self (i.e., a transpersonal or transcendent self), of others, and of the physical world (e.g., holism, nonmaterialism) during normal states of consciousness, accompanied by the capacity to identify their relationship to one’s self and to the physical world;
- Conscious State Expansion (CSE)—the ability to enter and exit higher/spiritual states of consciousness (e.g., pure consciousness, cosmic consciousness, unity, oneness) at one’s own discretion (as in deep contemplation or reflection, meditation, prayer, etc.).

As King (2008) claims, SI performs quite well according to the traditional criteria for intelligence as a phenomenon in the classic understanding (as explained in early twentieth-century literature). The above model satisfies the primary criterion: SI represents a set of mental abilities (as opposed to behaviors or experiences), and it has been empirically tested by King (2008). King’s approach is quite detailed and refined. Yet, additional research is needed to test SI’s empirical properties among different cultures. The original version of SISRI-24 demonstrated adequate validity and reliability, according to King, but more recent studies, even those with a large sample size (e.g., *n* = 834), do not consistently confirm King’s initial results (Atroszko et al. under review).

## Problems with Spiritual Intelligence and Its Importance

We can evaluate the above definitions of SI in conjunction with Gardner’s criteria for independent intelligence modalities using examples from the literature. In order to evaluate SI in light of the Gardner’s criteria, I adopt three basic assumptions

1. Spirituality is not identical to religiosity, but both have a common area that can be interpreted as spiritual religiousness or religious spirituality depending on the proportion of the two components (Skrzypińska 2014, 2016). The spiritual sphere of the individual is the basis for the implementation of beliefs, including religious ones; therefore, many phenomena related to religiosity (though not all) can be used to illustrate the spiritual activity of the sphere.
2. The basic motivation for the action of the spiritual sphere is the search for the meaning of life (thus the use of instruments facilitating this process).
3. Religion incites religiousness and all the consequences associated with it (e.g., religious behaviors: rites).

With these assumptions in mind, I explore SI in terms of Gardner’s criterion to assess whether it qualifies as a functional intelligence modality. Gardner (1993, chapter 4.) requires the following items of an independent intelligence modality.

1. An identifiable core operation or set of operations.
2. An evolutionary history and evolutionary plausibility.
3. A characteristic pattern of development.
4. Potential isolation by brain damage.
5. The existence of persons distinguished by the exceptional presence or absence of the ability.
6. Susceptibility to encoding in a symbol system.
7. Support from experimental psychological investigations.
8. Support from psychometric findings.

Fulfillment of the criterion 1 is possible if we assume that SI is an instrument for finding and discovering the meaning of life. These actions would be the core operation of SI. Obviously, if there is a need to indicate a detailed set of operations or specific actions, those indicated by Emmons (1999, mentioned above) may be stipulated.

Religion’s, and thus spirituality’s, place in evolutionary history (criterion 2) is documented in Boyer’s (2001) and McNamara (2006a) publications. Also, Jaynes (1976/2000), in his writings on the hypothesis of the bicameral mind, presents arguments that tightly relate the evolutionary plausibility of a religious understanding of reality to human consciousness.

Using Piaget’s (1972) stages of cognitive development and Fowler’s (1981) theories of faith development and stages of faith, we are able to reproduce a characteristic pattern of spiritual/religious development (criterion 3). Assuming, therefore, that the development of faith and religiosity are closely related to cognitive development (Fowler 1981; Ozorak 2005) and that the spiritual sphere is the basis and condition of this development (Paloutzian and Park 2013; Skrzypińska 2014, 2016), SI also goes through the same stages.

Criterion 4 could be verified according the suggestions made by Zohar and Marshall (2000). These authors indicate evidence of a so-called God spot becoming visible in the temporal lobe during spiritual activity. Neurotheology describes many examples of brain functioning during meditation or prayer as discovered in fMRI studies. The God Gene hypothesis goes further indicating that human spirituality is influenced by heredity. It is possible thanks to the presence of a specific gene [called vesicular monoamine transporter 2 (VMAT2)] which predisposes humans toward spiritual or mystic experiences (Hamer 2005).

With regard to the existence of persons distinguished by the exceptional presence or absence of SI (criterion 5), many scholars cite individual differences in the ability to search for the meaning of life (Park 2013) and the closely related ability to utilize spiritual resources to solve problems (Emmons 2000a). Some people are not able to comprehend a purpose of life or to find the meaning of life (perhaps due to absence of SI), that is why they register a low degree of well-being. Other individuals are able to identify the meaning of life part of the time, while still others are able to recognize the meaning of life at all times. Consequently, these latter individuals register a higher degree of well-being.

Religion and spiritual traditions serve as perfect examples of phenomena that use a well-structured symbolic code (criterion 6). If we assume that religion is a “product” of our spiritual sphere (as stated in the assumptions above), then SI is the instrument of our spiritual sphere that engages religious symbols to discover the meaning of life. SI is able to encode in a symbol system–such as those of language, numbers, graphics, or musical notation. The histories of different societies provide evidence for the creation of

- spiritual languages spoken by believers (e.g., *transcendence*, *eternity*, *nirvana*, *heavens*, etc.),
- magic numbers (e.g., *one* suggests uniqueness, an indivisible whole, or both; the words *Holy Trinity* indicate one god in three forms: *Omne trinum perfectum*; *666* as evil, etc.),
- symbolic graphics (e.g., the visualization of gods or demons, representations of the holy spirit, menorah, om, yin and yang, the ouroboros, etc.), and
- special kinds of music (e.g., Gregorian chant, Gospel music, Qawwali, vocalizations, Christian rock, etc.).

These are not only religious examples, but they are direct expressions of spirituality. SI can use them to initiate the capacity for transcendence, to enter into heightened spiritual states of consciousness, and to perform other activities mentioned by Emmons (2000b) in the process of solving existential dilemmas and finding the meaning of life.

Support from experimental psychological investigations (criterion 7) is not as extensive as might be expected. Criterion 7 is somewhat dependent on criterion 8, which should be fulfilled first to ensure that we are able to achieve reliable data during the process of measurement of SI. If we have good psychometric instruments, we will be able to project methodologically correct experiments. Criterion 7 is the biggest challenge for scholars nowadays.

The last criterion Gardner identifies, criterion 8, concerns support from psychometric findings. New questionnaires are aiding in the development of this area. We are still in the early stages, but tools such as SISRI-24 by King (2008), ISIS by Amram and Dryer (2008) or SSI by Kumar and Mehta (2011) are examples of research on SI that have resulted in specific findings.

Based on the above observations, SI meets most of the eight criteria Gardner deems necessary in order to qualify as an intelligence modality. But, even if SI does not meet all of Gardner’s criteria at this moment, does it necessarily follow that SI is not eligible to qualify as an intelligence modality? In the above statements, the properties SI exhibits do not conflict with the criteria as described by Gardner, and it is possible that SI may satisfy the remaining standards; perhaps the evidence has simply not yet been documented.

Gardner’s criteria stipulate very strict requirements for intelligence modalities. Emmons (1999, pp. 167–169) develops arguments illustrating a means by which SI mostly fulfills them. Not all scholars, however, agree with Gardner’s theories. Bruner (1983) responded to Gardner’s ideas, claiming that the intelligences were “useful fictions.” In Bruner’s opinion Gardner’s approach is far “beyond the data-crunching of mental testers.” Similarly, Gottfredson (2006) indicates that while thousands of studies support the importance of the intelligence quotient (IQ), empirical evidence for non-*g* intelligences is either lacking or very poor.

Gardner (Education Encyklopedia. 2017) did not want to accept the idea of SI, but instead suggested that ExI (also proposed in his research) may be a viable alternative construct. As Gardner (1993) clearly maintains: “Somewhat to my surprise, existential intelligence qualifies well as an intelligence in light of the eight criteria that I have set forth in my writings” (1993, chap. 4). This kind of phenomenon provides *sensitivity and capacity to tackle deep questions about human existence, such as the meaning of life, why do we die, and how did we get there* (Gardner 1999). Such an understanding carries a more general notion than the idea of SI proposed by other scholars: these existential questions are both broader and more basic. The SI construct emphasizes specific “tools” that are useful in solving the above mentioned problems, and moreover, SI is indispensable to the process of looking for the meaning of life (see King 2008). This last aspect is a crucial manifestation of evolutionary adaptation: without meaning, life is empty and devoid of purpose.

If Gardner is not certain that the precise number of intelligences has yet been determined, and he does not have confidence that the intelligences can be identified through statistical analyses of cognitive test results, then his theory still needs more evidence, as does the theory of SI. A long empirical road lies before scholars researching these intelligence models. There is a need for cross cultural research that contributes to our understanding of the universality of these concepts. Therefore, every study that seeks the real essence of these concepts is both valuable and challenging—even studies that create new questionnaires and/or adapt existing ones.

As Gardner (2000, p. 27) claims, “whether spirituality should be considered an intelligence depends upon definitions and criteria.” I consider spirituality as a dimension of personality (Emmons 1999; Piedmont 1999; MacDonald 2000; Skrzypińska 2014). As such, spirituality uses its motivation and potentiality to employ SI as an instrument to search for the meaning of life. It is not probable that the other types of intelligence (e.g., rational, emotional) would be able replace SI in this endeavor. There are many examples of people with high IQs (genius) who felt lost or lacking a sense of direction or purpose. Despite their excellent potential in one modality of intelligence, their experience of life was affected by their limited capacity for the effective existential reflection that can initiate the process of using SI to look for the meaning of life.

In light of the facts described above, one of the most important tasks for researcher is to study the relationship between *rational intelligence* (RI) and SI. King (2008) found no significant correlations between IQ and *spirituality quotient* (SQ^1^), indicators of RI and SI, respectively. On the basis of this finding, we can derive a general and workable hypothesis about the independence of SI and RI, which should be verified empirically, of course. This is a crucial clue concerning the assumption about the separate mechanisms that use different kinds of intelligence. We may suspect that RI is not the only adaptive instrument of human beings, because people with a high IQ do not always find meaning the meaning of life; sometimes they do not even cope with existence.

It comes as no surprise that SI correlates with EI. This has been demonstrated in a number of studies (e.g., Amram 2009; Chin et al. 2012; Dastjerdi et al. 2013). It is likely that these two types of intelligence cooperate with each other because, as in the case of finding the meaning of life, managing emotions is very important. Yet, Martin and Hafer (2009) did not find supportive data for the five models that Tischler et al. (2002) used to explain the relations among emotional intelligence, spirituality, and workplace performance. The latter authors believed that increasing competence is associated with personal awareness, personal skills, social awareness, and social skills. Again, the results of these studies indicate a need for additional research. It may be that the relations among these phenomena are more complicated than scholars at first supposed.

The pattern of the development of the intelligence modality across the lifespan may be another feature that distinguishes between RI and SI. We need a tool that will help us to find the meaning of life throughout our lives and not only for one part of it. IQ develops dynamically until about 16 years of age; then its rate of growth drops significantly; its development is limited mainly to youth. SQ appears to differ from IQ in this respect. King found that age was mildly related to SI and its subscales, lending potential support to the idea that SI develops throughout the lifespan (King 2008). This may be construed as evidence indicating that we are dealing with different types of abilities. All of these observations would benefit from verification through a series of studies.

## Spiritual Intelligence in Relation to Other Factors

The way a particular phenomenon relates to other variables serves as an indirect indicator of its function. Analysis of the relation may suggest the need for further verification, or a possible causal link. Does the relation operate as a predictor, or does the particular item, itself, hold the role of predictor? Analyzing various correlations provides a good starting point from which to make additional hypotheses. I will look at three such correlations in this section, relating SI to (a) personality, spirituality and existential intelligence; (b) to meaning of life and other phenomena related to spirituality; and (c) to well-being.

## Spiritual Intelligence in Relation to Personality, Spirituality, and Existential Intelligence

As I have stated above, one of the main assumptions in the theoretical approach taken by this article is that spirituality appears as a sixth dimension of personality in a context of Big Five Model (Emmons 1999; Piedmont 1999; MacDonald 2000; Skrzypińska 2014). Several researchers have demonstrated relations between these phenomena (e.g., MacDonald 2000), but they are weak, which may be a premise for seeking the relative independence of these constructs.

Using Big Five Model scholars have investigated SI as a factor of personality. Amrai et al. (2011) revealed its positive connections to conscientiousness, agreeableness, and extraversion, while also noting its negative connections to neuroticism (*N* = 205). Similarly, Madalaimuthu and Kadhiravan (2016), by means of regression analysis, proved that SI accounts for 18% of the variance in extraversion, 5% of the variance in agreeableness, 11% of the variance in conscientiousness, and 6% of the variance in emotional stability (*N* = 136). These results allow us to generate suppositions about the supportive role of SI in the process of utilizing some personality traits. Such a state of affairs could be a sign that SI uses the personality as an instrument for its purposes. Of course, in an empirical study it would be necessary to prove this causal sequence.

**Fig. 1:**
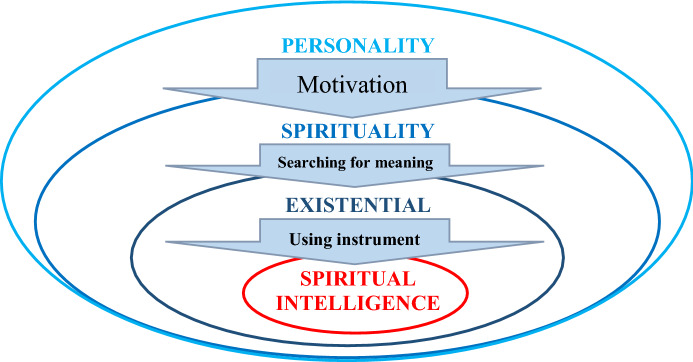
Theoretical model illustrating the relationship between personality and spirituality as well as existential and spiritual intelligence in a process of searching for the meaning of life

Summing up the previous part of the arguments, we propose a theoretical model (Fig. 1) that illustrates the relation between personality, spirituality, existential intelligence and spiritual intelligence. It seems that personality is of paramount importance in the process of creating the inner motivation that triggers the innate, human tendency to search for the meaning of life. At this stage, the personality engages its dimension of spirituality as the area responsible for building the meaning and purpose of life. Then spirituality activates existential intelligence as a means of developing a system of beliefs and values (Halama and Striženec 2004), or a sensitivity and capacity to tackle deep questions about human existence (Gardner 1999) either of which are useful in discovering the meaning of life. At the last stage, spiritual intelligence is used in direct actions aimed at real implementation of the intended goals.

Note that in the previous stage, existential intelligence triggers the generation of questions during the development of human consciousness, and it is only in the last stage that SI controls the process and provides tools such as the scales described by King (2008, and listed above): CET, PMP, TA, and CSE. Using SI in all its manifestations, the seeking person creates his/her own reality in a way that makes sense of his/her actions. Moreover, SI could be related to other capabilities and abilities that facilitate and enrich the process of looking for the meaning of life—especially in an adaptive context.

Other scholars, in addition to King, show interest in SI and its relation to other adaptive variables that are controlled by personality. One of the most cited of these factors is mindfulness. The results presented by Salmabadi et al. (2016) indicate the direct effects of mindfulness and SI on resiliency; and the direct effect of mindfulness on SI is also meaningful (*N* = 120). Scholars frequently study these relations in a workplace or other organizational setting. Subramaniam and Panchanatham (2015) revealed a statistically significant positive relationship between SI and mindfulness among employees in a workplace setting (*N* = 97). Gieseke (2014), who also conducted research in a workplace setting, proved a statistically significant positive relation among SI, mindfulness, and transformational leadership. These results illustrate what a useful and practical variable SI is and show how it could play crucial role as a mediator between other adaptive factors. Maybe mindfulness, as a state and process, can coordinate or even develop the capabilities related to SI? Mayer (2000) even claims that we should not consider using *spiritual intelligence* but rather *spiritual consciousness*. It seems, however, that one cannot equate consciousness with intelligence, because consciousness functions to recognize the processes that are happening (e.g., searching for the meaning of life), whereas SI is used to carry out such processes.

Although the above-described results are mixed and do not indicate a clear valence, the relationship between stress and SI is always negative (e.g., Du et al. 2013; Safavi et al. 2014; Salmabadi et al. 2015)*. Such a result illustrates that if SI is growing, stress levels drop. And this is a very important argument* taking this hint into the design of tests that will explore cause and effect relationship.

## SI in Relation to Meaning of Life and Other Phenomena Related to Spirituality

SI cannot be omitted as a link in the chain of psychic phenomena and events that lead to discovering the meaning of life. Although Emmons’ (1999) idea focuses on the theoretical implications of SI, other psychological literature indicates its importance and its relation to several crucial variables. Some of these pieces, with a stronger applied emphasis and adaptive character, are identified below.

One of the ways of presenting this type of complexity is to present the correlation of the appropriate measurement tools. King (2008) verified his SISRI-24 by correlating it with other questionnaires to achieve proper data related to validity. The following psychological measures were employed in order to validate and investigate the SISRI-24 (c.f. King 2008):

- Meaning in Life Questionnaire (MLQ; Steger et al. 2006),
- Metapersonal Self-Construal Scale (MSC; DeCicco and Stroink 2007),
- Mysticism Scale—Research Form D (MSD; Hood 1975),
- Age Universal Intrinsic–Extrinsic Religiosity Scale (AUIE; Gorsuch and Venable 1983),
- Satisfaction with Life Scale (SLS; Diener et al. 1985),
- Balanced Inventory of Desirable Responding (BIDR; Paulhus 1984)—social desirability,
- Emotional Intelligence Scale (EIS; Schutte et al. 1998),
- Multidimensional Aptitude Battery-II (MAB-II; Jackson 1998)—IQ.

Specifically with regard to the meaning of life, King indicated that the PMP scale was highly correlated with the Presence of Meaning, but not correlated with Search for Meaning. He found that CET was more highly correlated with Search for Meaning. This last finding could indicate that the act of critically contemplating meaning goes hand in hand with the actual search for meaning. The correlation displays the reflexive, conscious construction of meaning (c.f. Mayer 2000).

SI (represented by the all scales of SISRI-24) was highly correlated with Mysticism (MSD) and Metapersonal Self-Construal (MSC—the scale identifies the sense of one’s identity that extends beyond the individual or personal to encompass wider aspects of humankind, life, psyche, or the cosmos; Walsh and Vaughan 1993). SI was also found to be more significantly related to intrinsic religiosity than extrinsic religiosity. These findings provide evidence for a good theoretical fit of the measured constructs. As the literature points out, mysticism is very well correlated with spirituality (Streib and Hood 2016), and with the meaning of life (Griffiths et al. 2008).

## SI in Relation to Well-Being

SI is assumed to have adaptive function and a relation to better health and well-being. This is one of the most important reasons to further explore the SI phenomenon. King and DeCicco (2009) reported a relation between SI and Satisfaction with Life (*N* = 268), SI and Personal Meaning Production (.41), and SI and Transcendental Awareness (.21). Although these are only correlations, they indicate the potential for a directional relationship that may be present and would be worth seeking. The literature suggests that SI is an instrument launched in the spiritual sphere by PMP scale as a means of implementing well-being, and hence, health (Skrzypińska 2018). This can also happen when TA is understood as the capacity to identify transcendent dimensions/patterns of the self, of others, and of the physical world (Skrzypińska 2018), which probably is a condition for inspiring reflection that prompts individual to search for the meaning of life. King (2010) has reported that PMP appears to be highly adaptive in crises of an existential or spiritual nature, as well as in problems related to physical and psychological health. So its power cannot be neglected.

The literature provides examples of a specific measurement of the relationship between life satisfaction and SI. Kalantarkousheh et al. (2014) demonstrated a relevant relation between life satisfaction and SI among married and unmarried females (*N* = 202). There was a relation between life satisfactions in these two groups; however, there was no difference in terms of SI in these two groups. The results of a regression analysis have shown that SI is predictive of life satisfaction. Nevertheless, even in this area, inconsistent results appear. Dastjerdi et al. (2013) researched 123 gifted females and ascertained no meaningful correlation between SI and satisfaction, but they did find that emotional intelligence was related to satisfaction. In the workplace setting, Koražija et al. (2016) found no significant relationship between SI and work satisfaction for leaders, but they did find a significant, positive relationship between SI and workplace satisfaction for employees (*N* = 200).

Many findings also reveal no direct relationship between SI and satisfaction. Bigdeloo and Bozorgi (2016) have indicated that SI and self-control, together, can predict life satisfaction in high school teachers. Likewise, Munawar and Omama Tariq (2018) reported a significant correlation between spiritual intelligence, religiosity, and life satisfaction among elderly Pakistani Muslim people. In contrast to all of the aforementioned results, Koohbanani et al. (2013) proved a lack of relation between SI and satisfaction. It is only when SI and emotional intelligence are grouped together that they have a meaningful relationship with satisfaction. These findings illustrate that associations between SI and the cognitive component of well-being may not be simple, or direct. It is interesting that the internet includes many articles devoted to connections between SI and EI (emotional intelligence), but it is difficult to find SI’s relation to the emotions themselves, as components of well-being. Considering the regulatory role of emotions in human life, this seems to be a significant gap in scholarship.

Consequently, attempting to verify empirically the role of SI as a mediator, both between looking for the meaning of life and well-being (grasped as a whole, taking into account its cognitive and emotional component), and between looking for the meaning of life and health, is a worthy undertaking.

## Conclusion

Can we unquestionably say that SI exists and that it plays an essential role in the sphere of spirituality, helping in the process of searching for the meaning of life? The literature presents us with many reports on SI and accounts of its links with ExI. However, there is no empirical evidence that can actually and unambiguously establish a coherent model that explains the participation of SI in the process of looking for the meaning of life.

The human ability to find the meaning of life with the engagement of SI remains a complex puzzle that is still unsolved in terms of a psychological model. We should remember that the development of self-reflection, self-consciousness (Mayer 2000), and existential reflection can play a key role in searching for the meaning of life. Thus, an appropriate instrument for the verification of SI is needed. This should be the main goal of the next study. Therefore, I propose to verify the presented suppositions in two steps (studies): (a) we plan an adaptation and validation of King’s (2008) *Spiritual Intelligence Self*-*Report Inventory (SISRI*-*24)* to use in different countries (where SI has not yet been studied) to discover if SI exists cross culturally, as a universal phenomenon, (b) we intend to compare the relationships among the three kinds of intelligence modalities (rational, emotional, and spiritual) with reference to personality (including spirituality) and the search for the meaning of life. Following these studies, a series of experiments could be designed to evaluate the relations among the phenomena described in this article, with the aim of determining the nature and direction of any causal relations. These treatments would bring us closer to understanding the nature of human intelligence.

## Footnotes

1 Zohar and Marshall (2000) explain SQ as ‘our most fundamental intelligence. It is what we use to develop our capacity for meaning, vision and value. It allows us to dream and to strive. It underlies the things we believe in and the role our beliefs and values play in the actions that we take. Spiritual intelligence explores how accessing our SQ helps us to live up to our potential for better, more satisfying lives’.
